# MolViewSpec: a Mol* extension for describing and sharing molecular visualizations

**DOI:** 10.1093/nar/gkaf370

**Published:** 2025-05-06

**Authors:** Adam Midlik, Sebastian Bittrich, Jennifer R Fleming, Sreenath Nair, Sameer Velankar, Stephen K Burley, Jasmine Y Young, Brinda Vallat, David Sehnal

**Affiliations:** Protein Data Bank in Europe, European Molecular Biology Laboratory, European Bioinformatics Institute, Hinxton, Cambridge CB10 1SD, United Kingdom; Research Collaboratory for Structural Bioinformatics Protein Data Bank, San Diego Supercomputer Center, University of California, La Jolla, CA 92093, United States; Protein Data Bank in Europe, European Molecular Biology Laboratory, European Bioinformatics Institute, Hinxton, Cambridge CB10 1SD, United Kingdom; Protein Data Bank in Europe, European Molecular Biology Laboratory, European Bioinformatics Institute, Hinxton, Cambridge CB10 1SD, United Kingdom; Protein Data Bank in Europe, European Molecular Biology Laboratory, European Bioinformatics Institute, Hinxton, Cambridge CB10 1SD, United Kingdom; Research Collaboratory for Structural Bioinformatics Protein Data Bank, San Diego Supercomputer Center, University of California, La Jolla, CA 92093, United States; Research Collaboratory for Structural Bioinformatics Protein Data Bank and the Institute for Quantitative Biomedicine, Rutgers, The State University of New Jersey, Piscataway, NJ 08854, United States; Rutgers Cancer Institute, Rutgers, The State University of New Jersey, New Brunswick, NJ 08901, United States; Department of Chemistry and Chemical Biology, Rutgers, The State University of New Jersey, Piscataway, NJ 08854, United States; Rutgers Artificial Intelligence and Data Science Collaboratory, Rutgers, The State University of New Jersey, Piscataway, NJ 08854, United States; Research Collaboratory for Structural Bioinformatics Protein Data Bank and the Institute for Quantitative Biomedicine, Rutgers, The State University of New Jersey, Piscataway, NJ 08854, United States; Research Collaboratory for Structural Bioinformatics Protein Data Bank and the Institute for Quantitative Biomedicine, Rutgers, The State University of New Jersey, Piscataway, NJ 08854, United States; Rutgers Cancer Institute, Rutgers, The State University of New Jersey, New Brunswick, NJ 08901, United States; Protein Data Bank in Europe, European Molecular Biology Laboratory, European Bioinformatics Institute, Hinxton, Cambridge CB10 1SD, United Kingdom; National Centre for Biomolecular Research, Faculty of Science, Masaryk University, Brno 625 00, Czech Republic

## Abstract

Data visualization is a pivotal component of a structural biologist’s arsenal. The Mol* Viewer makes molecular visualizations available to broader audiences via most web browsers. While Mol* provides a wide range of functionality, it has a steep learning curve and is only available via a JavaScript interface. To enhance the accessibility and usability of web-based molecular visualization, we introduce MolViewSpec (molstar.org/mol-view-spec), a standardized approach for defining molecular visualizations that decouples the definition of complex molecular scenes from their rendering. Scene definition can include references to commonly used structural, volumetric, and annotation data formats together with a description of how the data should be visualized and paired with optional annotations specifying colors, labels, measurements, and custom 3D geometries. Developed as an open standard, this solution paves the way for broader interoperability and support across different programming languages and molecular viewers, enabling more streamlined, standardized, and reproducible visual molecular analyses. MolViewSpec is freely available as a Mol* extension and a standalone Python package.

## Graphical Abstract

**Graphical Abstract:**
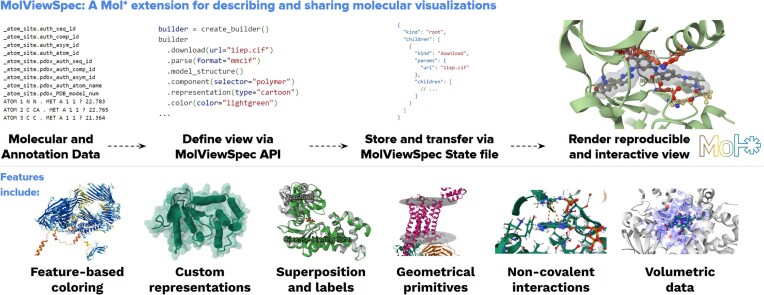

## Introduction

The Protein Data Bank (PDB) [1] has been the primary repository for experimentally determined three-dimensional, atomic-level biostructures for over 50 years and has been fundamental in elucidating molecular functions and interactions. The archive currently contains >235 000 3D structures of macromolecules and their complexes. The PDB repository is managed according to the Findable, Accessible, Interoperable, and Reusable (FAIR) Principles [2] by the Worldwide Protein Data Bank partnership (wwPDB; wwpdb.org) [1, 3], an international consortium that collaboratively oversees deposition, validation, biocuration, remediation, and open access dissemination of 3D macromolecular structure data. This information is vital for understanding processes that make life possible, because function follows form in biology. Moreover, the landscape of structural biology has been transformed with the emergence of novel computational prediction methods that have been developed using open-access PDB data [4]. New generation AI-powered tools like AlphaFold [5], ESMFold [6], and OpenFold [7] provide access to millions of predicted structures.

One of the primary methods of interacting with 3D molecular structures is by visualizing them using molecular graphics viewers such as Mol* [8], JalView [9], and ChimeraX [10]. Furthermore, tools like Protein Imager [11] provide an easy-to-use web interface for users to conveniently create macromolecular visualizations. These tools offer a wide variety of functionalities and are essential for visually inspecting and analyzing the details of macromolecular structures. 3D visualization of molecular structures provides insights into the spatial arrangement, interactions, and shapes of molecules, which are crucial for understanding their functions, properties, and behaviors in biological systems. Enriching 3D biostructures obtained from experimental or calculated sources with biological and functional annotations is essential for data interpretation, allowing scientists to unlock immense value for both research and education. At present, this is achieved by collecting this information from the many available data resources (often manually) and then displaying it on structures within graphics visualization software. This process is cumbersome and limits scalability and reproducibility. Therefore, facile and reproducible methods for visualizing 3D structures of macromolecules and related annotations are essential for increasing their utilization and enabling robust analysis and interpretation of 3D biostructure information by millions of users worldwide.

Mol* is a widely used software that enables the visual analysis of macromolecular structures via the web browser. Although Mol* offers extensive functionality, it has a steep learning curve and is limited to a JavaScript interface. To make web-based molecular visualization more accessible and user-friendly, we have developed MolViewSpec (molstar.org/mol-view-spec)—a standardized approach for defining molecular visualizations that separates the creation of complex molecular scenes from viewer-specific details. A prototype implementation of MolViewSpec, demonstrating reproducible visualization protocols, was previously published [12]. In this work, we highlight the full range of MolViewSpec capabilities, along with new features and expanded functionalities that have been incorporated based on community feedback. These features enable the creation of compelling visual narratives, as demonstrated in later sections. MolViewSpec is available both as an extension to Mol* and as a standalone Python-based toolkit, serving users outside of the JavaScript ecosystem. The open standard specified by MolViewSpec allows for implementation in additional programming languages and adoption by other visualization tools, thus promoting interoperability and enabling a seamless visualization experience for users.

MolViewSpec development and maintenance are a collaborative effort by Protein Data Bank in Europe (PDBe), Research Collaboratory for Structural Bioinformatics Protein Data Bank (RCSB PDB) in the United States, and National Centre for Biomolecular Research (NCBR)/Central European Institute of Technology (CEITEC) in the Czech Republic.

The following sections describe MolViewSpec’s functionality, architecture, and implementation, demonstrated using two examples.

## Description of the service

### Functionality

MolViewSpec establishes a standardized declarative, data-driven method to describe 3D molecular scenes. We refer to these scenes as MolViewSpec States. These can encompass one or more molecular structures enriched by biologically relevant annotations. Specifically, MolViewSpec offers the following functionalities:

- Specifying data source and format of 3D molecular structural data with focus on the PDBx/mmCIF [13], BinaryCIF [14], and PDB [15] formats.
- Constructing molecular structures, including the specification of asymmetric unit, biological assembly, or crystal symmetry.
- Defining selections via predefined structure components (protein, ligand, nucleic acids, carbohydrates [16], etc.) or lists of atoms, residues, chains, or molecular entities.
- Specifying 3D molecular representations (cartoons, ball and stick, surface, etc.).
- Volumetric data rendering (isosurface).
- Labeling and coloring individual selections, facilitating visualization of annotations.
- Specifying structure coordinate transformations to enable structural superposition.
- Visualization of geometrical primitives (meshes, lines, spheres, and distance/angle measurements, etc.).
- Controlling camera position within the scene, allowing either a static viewpoint or automatic focus on a specific selection.
- State transition between multiple views to create visual narratives.

**Figure 1.**
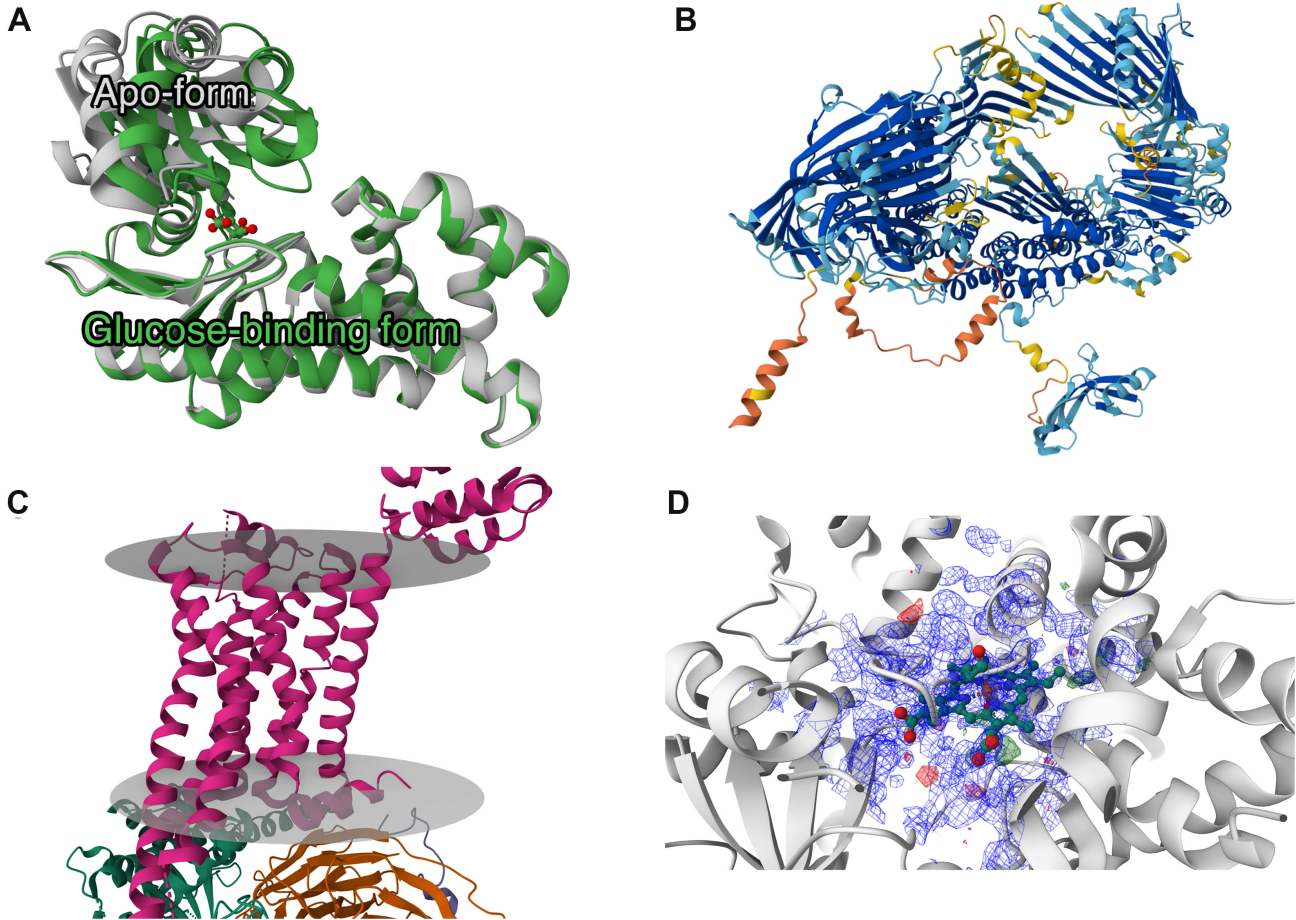
Illustration of selected MolViewSpec features. (**A**) apo-form [PDB ID 2e2n, chain A] and the glucose-binding form [PDB ID 2e2o, chain A] of the ATP-dependent hexokinase from *Sulfolobus tokodaii* [18], superposed based on its the largest domain. (**B**) An AlphaFold predicted structure (UniProt ID Q868N5) colored by residue-wise predicted Local Distance Difference Test (pLDDT) confidence score [19]. (**C**) Predicted membrane orientation for the β_2_ adrenergic receptor–Gs protein complex (PDB ID 3sn6 [20]). (**D**) Electron density map around a ligand (HEM, PDB ID 1tqn [21]). See Supplementary Materials 2–5 for source code of these examples. More examples are available at molstar.org/mol-view-spec.

The basis for MolViewSpec functionalities (see Fig. 1 for illustration of selected features) has been tailored to facilitate the creation of reproducible and interactive visualizations akin to those found in data resources like PDB and AlphaFold database [17]. This empowers a broader audience to leverage these visualizations effectively, simplifying and streamlining the visualization process. Unlike static images often used in publications, MolViewSpec enables dynamic exploration and manipulation, allowing users to delve deeper into the data and gain richer insights.

MolViewSpec integrates with the Mol* Viewer through a dedicated extension, providing visualization functionality that can be accessed in a variety of ways:

- Directly loading MolViewSpec State in the Mol* Viewer, either by drag-and-drop or via the menu in the viewer’s graphical interface.
- Providing a link to or embedding a MolViewSpec State as part of the browser URL.
- Using JavaScript to either specify or load the MolViewSpec State into a Mol* Viewer instance embedded in external applications.
- Command-line utility that enables rendering of MolViewSpec States as static PNG or JPEG images, eliminating the need for a web browser.

See Supplementary Materials 1 and 6 and the MolViewSpec documentation available on the MolViewSpec webpage for more information on how to access this functionality.

### Architecture and implementation

#### Architecture

**Figure 2.**
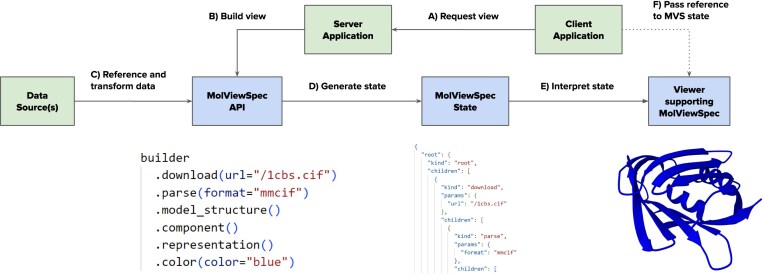
MolViewSpec architecture. (**A**) The client application requests a view from a server, (**B**) the server utilizes the MolViewSpec API to (**C**) reference and/or transform data sources and (**D**) generate MolViewSpec State (encoded as a JSON object), which (**E**) can be interpreted by a viewer; (**F**) the client application passes the MolViewSpec State to the viewer. Steps (B)–(D) can be replaced by serving a pre-generated static MolViewSpec State file instead of generating it dynamically.

The purpose of MolViewSpec is to provide a mechanism for specifying reproducible 3D molecular scenes (MolViewSpec States). To achieve this, it defines a structured data representation organized in a nested tree format that contains all information necessary to reproduce a 3D molecular visualization. The MolViewSpec State data structure is stored using the JavaScript Object Notation (JSON) format. Due to the widespread adoption of JSON, MolViewSpec States can be built using all mainstream programming languages and interpreted by a wide range of molecular viewers. This principle is illustrated in Fig. 2.

#### Implementation

The MolViewSpec toolkit currently consists of three parts:

- MolViewSpec State JSON Schema, which defines the properties of individual nodes of the tree data structure stored in files with MVSJ and MVSX extensions;
- MolViewSpec API that provides a mechanism for building and validating MolViewSpec States stored in JSON format defined by the Schema. The API is available in Python and JavaScript/TypeScript programming languages. This API can be implemented in any language supporting the JSON format (e.g. Java or C#);
- MolViewSpec Mol* Viewer extension implemented in TypeScript that provides the ability to interpret and render MolViewSpec States in a web browser environment. Different viewers could adopt the specification and support loading MolViewSpec States.

## Results and discussion

We demonstrate the capabilities of MolViewSpec through scientifically insightful examples, carefully selected to showcase the rich and unique functionality of our solution. The source code for these use cases is accessible via the MolViewSpec documentation.

### Structural stories of BCR-ABL kinase and TATA-binding protein

**Figure 3.**
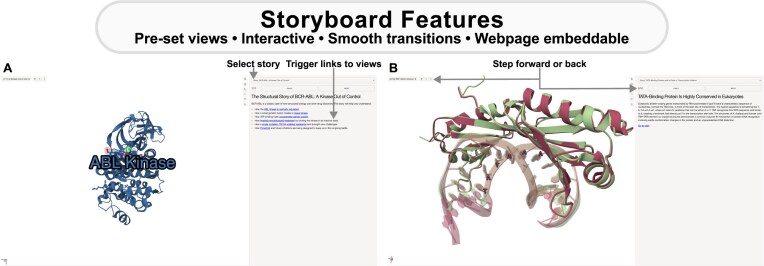
Structural stories of BCR-ABL kinase and TBP. MolViewSpec can be used to create interactive storyboards. We illustrate this by showing (**A**) how structural changes drive disease, influence structure-based drug discovery, and contribute to drug resistance in BCR-ABL kinase (PDB ID 1opl [24]) by visualising key molecular features such as the ATP-binding pocket, regulatory domains, and drug resistance-associated mutations in the kinase catalytic domain; and (**B**) the critical role of the highly conserved TBP in initiating mRNA transcription in eukaryotes by highlighting protein–DNA interactions resulting in subtle conformation changes in TBP and an unprecedented distortion in DNA (structural alignment of 1cdw [23] and 1vtl [25] shown). Explore the full stories at molstar.org/demos/mvs-stories. The application can be embedded into 3rd party web pages and used as a template to tell similar narratives.

We have used MolViewSpec to build interactive storyboards (see Fig. 3) that guide readers through the structure, function, and interactions of two important proteins: BCR-ABL kinase and TATA-binding protein (TBP). The BCR-ABL fusion kinase, resulting from the Philadelphia chromosome translocation, is responsible for chronic myeloid leukaemia [22], and serves as a compelling example of how structural changes drive disease, influence structure-based drug design, and contribute to drug resistance. The storyboard depicts its activation mechanism, inhibitor binding, conformational states, and escape mutations. The second story highlights the critical role of the highly conserved TBP in initiating messenger RNA (mRNA) transcription in eukaryotes [23]. Situated at the heart of the complex macromolecular machinery involved in RNA polymerase II transcription initiation, TBP plays a central role in DNA recognition and assembling the pre-initiation complex, as illustrated in the storyboard. These visual representations demonstrate how MolViewSpec facilitates the creation of interactive, structured storyboards by incorporating hyperlinks to preset molecular views and external resources, enabling users to explore key structural features and functional insights in an engaging and guided manner. Pre-defined views ensure that molecular representations are consistently oriented for clarity, facilitating an intuitive understanding of complex concepts. Smooth state transitions maintain visual context when zooming in on specific regions, helping users follow the narrative. Additionally, the labelling function enhances storytelling by providing clear annotations that highlight critical molecular features. Organizing molecular stories in an engaging and interactive way maximizes comprehension and impact, making it a valuable tool for conveying complex concepts with broad applications that include public outreach and science communication to education, training, and laboratory resources.

### Visualization of spatial restraints used in integrative modeling investigations

Recent advances in structural biology have led to the development of integrative and hybrid structure determination methods (IHM) [26] that combine information from one or more experimental techniques [e.g. chemical crosslinking mass spectrometry (crosslinking-MS) and 3D electron microscopy] with computational modeling algorithms. PDB-IHM (previously known as PDB-Dev) is a new branch of the PDB archive created to support deposition, curation, validation, and dissemination of integrative or IHM structures [27].

**Figure 4.**
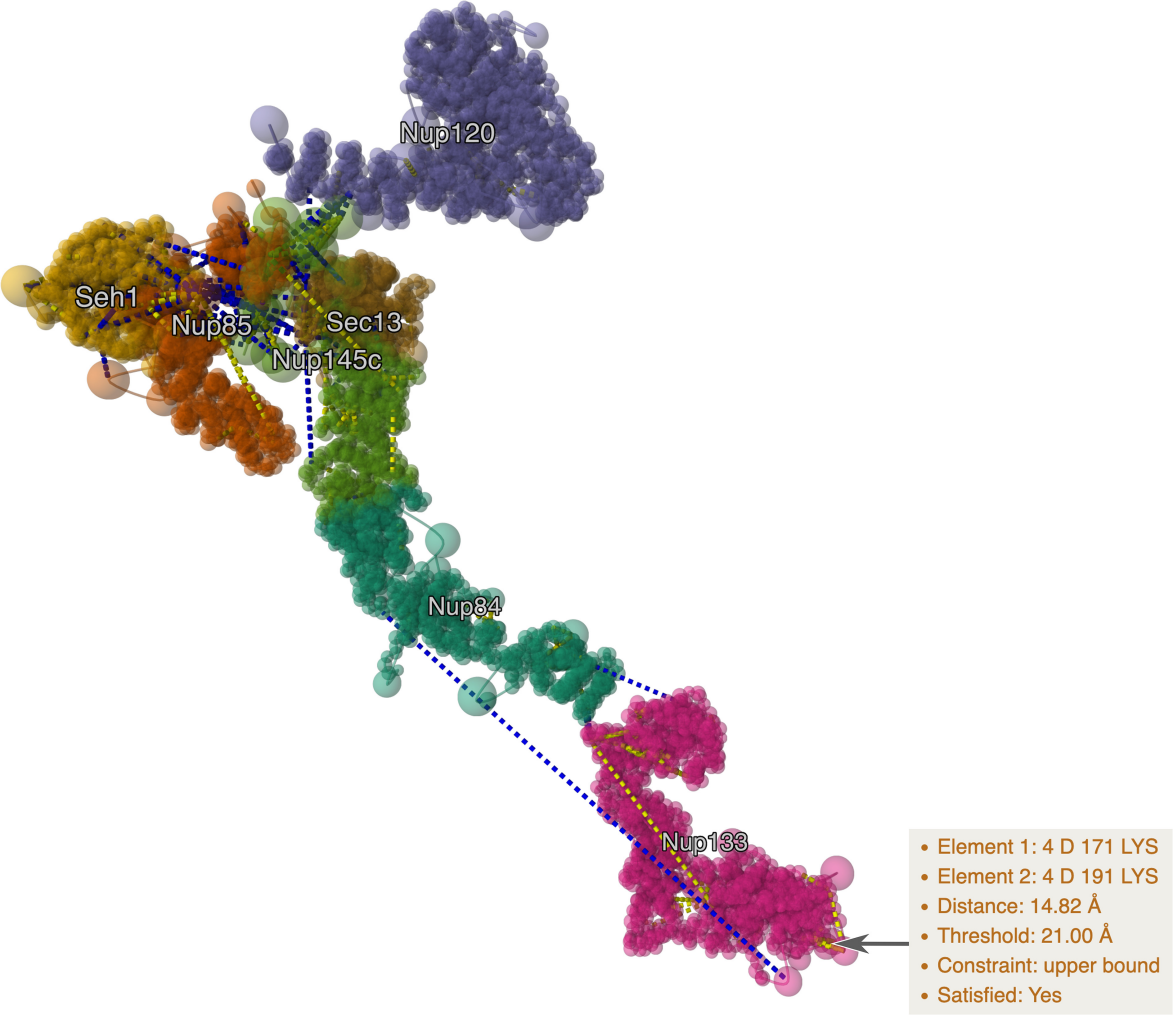
MolViewSpec-guided Mol* illustration of a multiscale integrative structure and associated distance restraints from crosslinking mass spectrometry data. The structure of the Nup84 subcomplex of the Nuclear Pore Complex from budding yeast obtained from an integrative modeling [28] is shown. The structure is archived in the PDB-IHM sub-directory of the PDB (PDB ID 8zz1) and is based on input restraints from crosslinking-MS data and 2D electron microscopy class averages. The coarse-grained spherical beads at different granularities in the multiscale model are depicted. Individual subunits in the Nup84 subcomplex are identified using different colors and labels. The intra- and inter-subunit crosslink restraints used in the model are identified using dotted lines with an example crosslink highlighted and available as a tooltip. An interactive version is available at molstar.org/demos/ihm-restraints.

Restraints obtained from crosslinking-MS experiments have been widely used to determine integrative structures archived in PDB-IHM and are typically applied at the residue level as pairwise distances. Herein, we demonstrate MolViewSpec’s use to translate the crosslink restraint data stored in the mmCIF files to dynamically map and visualize these pairwise distances on the 3D coordinate data (Fig. 4). Using the arsenal of Mol* and MolViewSpec capabilities, this example illustrates the visual characterization of intra- and inter-molecular crosslinks. Such visual analysis can be powerful for interpreting model quality and for portraying model fit to the input experimental data for model validation.

### Limitations and outlook

With MolViewSpec, our goal is to provide standardized tools for molecular visualization, emphasizing interoperability and reproducibility while ensuring its ongoing development and extension. The initial version is designed for integration with the Mol* Viewer and includes a core feature set to support a wide visualization range within the PDB infrastructure. As an extensible standard, MolViewSpec continues to evolve, enabling further enhancements based on community needs.

Our future goals for MolViewSpec include:

- Actively addressing community requirements as they arise.
- Standardizing molecular visualization color schemes (currently user-defined).
- Supporting programming languages other than JavaScript/TypeScript and Python, e.g. Java or C#.
- Coordinating with developers of major 3D viewers like PyMOL and ChimeraX to facilitate MolViewSpec cross-platform support and enhance interoperability, allowing researchers to create consistent and reusable visualizations.
- MolViewSpec is currently designed primarily for developers. Creating a user-friendly interface for building MolViewSpec States will enhance its accessibility and broaden its utility.

## Conclusion

We introduce MolViewSpec, a tool for standardized descriptions of reproducible molecular visualizations. MolViewSpec enables a programming language agnostic way of describing molecular views, currently implemented in Python and JavaScript/TypeScript. This data-driven mechanism seamlessly integrates structural and volumetric data with annotations, representations, custom geometries, and state transitions, enabling the creation of reproducible visualizations. MolViewSpec is available as a Mol* extension that supports the creation and visualization of molecular view states and as a Python package that can be used via a declarative API. MolViewSpec eliminates the steep learning curve of Mol* and offers a more convenient, user-friendly, and accessible way to create visual narratives. The specifications and toolkit can be adopted by other visualization software and programming languages, promoting interoperability and providing streamlined, standardized, and reproducible visual scenes that facilitate robust analysis and interpretation of 3D molecular structures.

## Supplementary Material

gkaf370_Supplemental_Files

## Data Availability

The MolViewSpec toolkit and the Mol* extension are available online at molstar.org/mol-view-spec, including documentation. This website is free and open to all users with no login requirements. For developers and researchers interested in customization or further development, the complete source code is under the MIT license on GitHub (github.com/molstar/mol-view-spec, github.com/molstar/molstar/tree/master/src/extensions/mvs). MolViewSpec can be installed using Python’s package manager from the PyPi repository (pypi.org/project/molviewspec). A Colab Notebook (tinyurl.com/yc6zp8yu) with an interactive tutorial is provided to illustrate the toolkit capabilities and facilitate user familiarization. All source code at publication is archived on Zenodo (doi.org/10.5281/zenodo.15213735).
