# Supplementary material for: MolViewSpec: a Mol* extension for describing and sharing molecular visualizations
Source: Nucleic Acids Res. 2025 May 6;53(W1):W408–14. doi: 10.1093/nar/gkaf370 (PMC12230705; doi:10.1093/nar/gkaf370)
Supplement: gkaf370_Supplemental_Files [file gkaf370_supplemental_files.zip › Supplementary Material 1.pdf]

Supplementary Material 1: Loading MolViewSpec state into Mol* Viewer

This section provides details on different methods of loading a MolViewSpec state in the Mol* Viewer. Even more detailed, up-to-date information can be found in the official documentation (https://molstar.org/mol-view-spec-docs/).

1) Directly loading files in the Mol* Viewer (https://molstar.org/viewer) is the most straightforward method and is appropriate for loading MVSJ and MVSX files from a local disk. Either drag the file and drop it in a browser window with Mol* Viewer, or use the “Open Files” or “Download File” option in the left menu.

2) A link to a MolViewSpec file can be provided as part of the browser URL, by specifying `mvs-format` and `mvs-url` parameters. Examples of this include:
https://molstar.org/viewer/?mvs-format=mvsj&mvs-url=https://raw.githubusercontent.com/molstar/molstar/master/examples/mvs/1cbs.mvsj
https://molstar.org/viewer/?mvs-format=mvsx&mvs-url=https://raw.githubusercontent.com/molstar/molstar/master/examples/mvs/1h9t.mvsx

Alternatively, smaller files can be directly inlined in the browser URL using the `mvs-data` parameter (see documentation for examples). This removes the necessity for hosting the file; however, because of the URL length limit, this will not work for larger files. The preferred method is to use `mvs-url`.

3) Another approach is to use JavaScript to load a MolViewSpec state into a Mol* Viewer hosted by a 3rd party application. The following HTML snippet creates a Mol* Viewer instance, fetches a MVS file, and loads it in the Mol* Viewer instance:

```
Unset
<!DOCTYPE html>
<html lang="en">


<head>
```

```
    <script
src="https://cdn.jsdelivr.net/npm/molstar@4.12.1/build/viewer/molstar.js"></scr
ipt>
    <link rel="stylesheet" type="text/css"
href="https://cdn.jsdelivr.net/npm/molstar@4.12.1/build/viewer/molstar.css" />
</head>

<body>
    <div id="viewer1" style="position: relative; width: 500px; height:
500px;"></div>
    <script>
        const sourceUrl =
'https://raw.githubusercontent.com/molstar/molstar/master/examples/mvs/1h9t_dom
ain_labels.mvsj';
        molstar.Viewer.create('viewer1', { layoutIsExpanded: false,
layoutShowControls: false })
            .then(viewer => viewer.loadMvsFromUrl(sourceUrl, 'mvsj'));
    </script>
</body>

</html>
```

More customizable and interactive integration can be achieved using the *MVSData* object (provides parsing, encoding, validating, and building MVS) and *loadMVS* function (provides visualization). With this approach, the MVS view can be obtained from a URL or can be built ad-hoc within the page's JavaScript code. See the documentation for more details and examples (https://molstar.org/mol-view-spec-docs/).

4) Lastly, Mol* provides a few command-line utilities, which can be executed via Node.js (version 18 or higher), most notably *mvs-render* to create PNG or JPEG images from MolViewSpec files (this functionality requires installing additional npm packages, most importantly the *gl* rendering library).
