# Supplementary material for: MolViewSpec: a Mol* extension for describing and sharing molecular visualizations
Source: Nucleic Acids Res. 2025 May 6;53(W1):W408–14. doi: 10.1093/nar/gkaf370 (PMC12230705; doi:10.1093/nar/gkaf370)
Supplement: gkaf370_Supplemental_Files [file gkaf370_supplemental_files.zip › Supplementary Material 2.pdf]

Supplementary Material 2: Defining Figure 1a

The following text describes how to create the MVS states presented in Figure 1a. The code snippets are written in TypeScript and use the builder interface provided in the Mol* library (equivalent Python code is provided below).

The first step is to create a MVS builder. The builder initially contains an empty state and provides methods for adding nodes to the state, each node representing an instruction to the viewer. To load a structure, we need to download the structure file first, then apply parsing to the downloaded file, and finally specify what structure should be read from the parsed file (in case there are multiple models, assemblies etc.):

JavaScript
```
import { MVSData } from 'molstar/lib/commonjs/extensions/mvs/mvs-data';

const builder = MVSData.createBuilder();
const structure1 = builder
    .download({ url: 'https://files.wwpdb.org/download/2e2n.cif' })
    .parse({ format: 'mmcif' })
    .modelStructure({});
```

This still does not create any visible objects. For that, we need to add 3D representation for the components of the structure. The representation can be further modified by applying colors:

JavaScript
```
structure1
    .component({ selector: { label_asym_id: 'A' } })
    .representation({ type: 'cartoon' })
    .color({ color: '#dddddd' });
```

Now the state contains a cartoon representation for the apo-form of the enzyme (PDB ID 2e2n). Next, we will load the structure of the glucose-binding form in a similar way and apply a transformation that superposes the large domains of the two structures:

```javascript
const structure2 = builder
    .download({ url: 'https://files.wwpdb.org/download/2e2o.cif' })
    .parse({ format: 'mmcif' })
    .modelStructure({})
    .transform({
        rotation: [
            0.291445, 0.949818, 0.113601,
            -0.479952, 0.042465, 0.876266,
            0.827469, -0.309906, 0.468243,
        ],
        translation: [2.237313, 17.994696, -4.031342],
    });
```

For the glucose-binding form, we will add two representations – cartoon for the protein itself and ball-and-stick for the ligand. We will also color the oxygen atoms of the ligand selectively:

```javascript
structure2
    .component({ selector: { label_asym_id: 'A' } })
    .representation({ type: 'cartoon' })
    .color({ color: '#4fc64f' });
structure2
    .component({ selector: { label_asym_id: 'B' } })
    .representation({ type: 'ball_and_stick' })
    .color({ color: '#4fc64f' })
    .color({ color: 'red', selector: { type_symbol: 'O' } });
```

Similarly, components can be used to add labels to the scene. The following example selects based on chain identifiers and also makes use of sequence ranges to position the labels accurately:

```JavaScript
structure1
    .component({
        selector: {
            label_asym_id: 'A', beg_label_seq_id: 1, end_label_seq_id: 50
        }
    })
    .label({ text: 'Apo-form' });
structure2
    .component({
        selector: {
            label_asym_id: 'A', beg_label_seq_id: 201, end_label_seq_id: 250
        }
    })
    .label({ text: 'Glucose-binding form' });
```

Last but not least, we set the camera position and orientation:

```JavaScript
builder.camera({
    position: [-4.449025, 31.275798, 17.857061],
    target: [49.825582, -1.340038, 26.471059],
    up: [-0.068663, -0.098933, 0.992722],
});
```

Now the state is ready and can be retrieved in MVSJ format:

```JavaScript
const mvsj = MVSData.toMVSJ(builder.getState(), 2);
console.log(mvsj);
```

The output of this code can be saved to a file with .mvsj extension and then simply loaded in Mol*.

Equivalent code in Python will look very similar:

```python
import molviewspec as mvs

builder = mvs.create_builder()

structure1 = (
    builder
    .download(url='https://files.wwpdb.org/download/2e2n.cif')
    .parse(format='mmcif')
    .model_structure()
)
(structure1
 .component(selector=mvs.ComponentExpression(label_asym_id='A'))
 .representation(type='cartoon')
 .color(color='#dddddd')
 )

structure2 = (
    builder
    .download(url='https://files.wwpdb.org/download/2e2o.cif')
    .parse(format='mmcif')
    .model_structure()
    .transform(
        rotation=[
            0.291445, 0.949818, 0.113601,
            -0.479952, 0.042465, 0.876266,
            0.827469, -0.309906, 0.468243,
        ],
        translation=[2.237313, 17.994696, -4.031342])
)
(structure2
 .component(selector=mvs.ComponentExpression(label_asym_id='A'))
 .representation(type='cartoon')
```

```
 .color(color='#4fc64f')
 )
(structure2
 .component(selector=mvs.ComponentExpression(label_asym_id='B'))
 .representation(type='ball_and_stick')
 .color(color='#4fc64f')
 .color(color='red', selector=mvs.ComponentExpression(type_symbol='O'))
 )
(structure1.component(selector=mvs.ComponentExpression(label_asym_id='A',
beg_label_seq_id=1, end_label_seq_id=50))
 .label(text='Apo-form')
 )
(structure2
 .component(selector=mvs.ComponentExpression(label_asym_id='A',
beg_label_seq_id=201, end_label_seq_id=250))
 .label(text='Glucose-binding form')
 )
builder.camera(
    position=[-4.449025, 31.275798, 17.857061],
    target=[49.825582, -1.340038, 26.471059],
    up=[-0.068663, -0.098933, 0.992722],
)

print(builder.get_state())
# Or save into a file directly:
# builder.save_state(destination='1a.mvsj', indent=2)
```
