# Supplementary material for: MolViewSpec: a Mol* extension for describing and sharing molecular visualizations
Source: Nucleic Acids Res. 2025 May 6;53(W1):W408–14. doi: 10.1093/nar/gkaf370 (PMC12230705; doi:10.1093/nar/gkaf370)
Supplement: gkaf370_Supplemental_Files [file gkaf370_supplemental_files.zip › Supplementary Material 3.pdf]

Supplementary Material 3: Defining Figure 1b

The following text describes how to create the MVS state presented in Figure 1b. The code snippets are written in TypeScript and use the builder interface provided in the Mol* library (equivalent Python code is provided below).

As with the previous example, we first need to create a MVS builder, download the structure file, apply parsing, and load the model structure:

```javascript
import { MVSData } from 'molstar/lib/commonjs/extensions/mvs/mvs-data';

const builder = MVSData.createBuilder();
const structure = builder
    .download({ url:
'https://alphafold.ebi.ac.uk/files/AF-Q868N5-F1-model_v4.cif' })
    .parse({ format: 'mmcif' })
    .modelStructure({});
const component = structure.component({ selector: 'all' });
```

In this example, we will only create one component, containing the whole structure. We can focus this component and specify camera orientation (we could also use `camera` instead of `focus`, as in the previous example):

```javascript
const component = structure.component({ selector: 'all' });
component
    .focus({
        direction: [-0.549278, 0.082181, -0.831588],
        up: [-0.087114, 0.984098, 0.154794],
    });
```

Now we can create a cartoon representation and apply coloring. Instead of specifying the colors for each residue in the MVS state file itself, we will reference a MVS annotation file that contains the data for coloring. The MVS annotation file can be formatted as JSON, CIF, or BCIF (BinaryCIF). In this case we use the file ‘confidence-Q868N5.cif’, which is included in the Supplementary Material 4.

```javascript
component
    .representation({ type: 'cartoon' })
    .colorFromUri({ uri: './confidence-Q868N5.cif', format: 'cif', schema: 'residue', category_name: 'confidence', field_name: 'color' });
```

Now the state is ready and can be retrieved in MVSJ format:

```javascript
const mvsj = MVSData.toMVSJ(builder.getState(), 2);
console.log(mvsj);
```

The output of this code can be saved to a file with .mvsj extension. However, when loading into Mol* we will need to ensure that the annotation file is available as well. This can be achieved by:

a) hosting the MVS state file and MVS annotation file together at the same server, so that the relative URI reference ‘./confidence-Q868N5.cif’ resolves to the URI of the annotation file (this approach is preferred when integrating MVS views in a web application);

b) packing the MVS state file and MVS annotation file together into an MVSX file (this can then be loaded to Mol* using drag-and-drop method). To create this MVSX file, we just create a ZIP archive containing the MVS state file (must be named ‘index.mvsj’) and ‘confidence-Q868N5.cif’, and change the archive’s file extension from ‘.zip’ to ‘.mvsx’.

Equivalent code in Python will look very similar:

```python
import molviewspec as mvs

builder = mvs.create_builder()

structure = (builder
 .download(url='https://alphafold.ebi.ac.uk/files/AF-Q868N5-F1-model_v4.cif')
 .parse(format='mmcif')
 .model_structure()
 )
(structure
 .component(selector='all')
 .focus(direction=[-0.549278, 0.082181, -0.831588],
        up=[-0.087114, 0.984098, 0.154794])
 .representation(type='cartoon')
 .color_from_uri(uri='./confidence-Q868N5.cif', format='cif',
                 schema='residue', category_name='confidence',
                 field_name='color')
 )

builder.save_state(destination='1b.mvsj', indent=2)
```

And the following Python code packs the MVS state file and MVS annotation file together into an MVSX file:

```python
with zipfile.ZipFile('1b.mvsx', mode='w') as z:
    z.write('1b.mvsj', arcname='index.mvsj')
    z.write('confidence-Q868N5.cif', arcname='confidence-Q868N5.cif')
```
