# Supplementary material for: MolViewSpec: a Mol* extension for describing and sharing molecular visualizations
Source: Nucleic Acids Res. 2025 May 6;53(W1):W408–14. doi: 10.1093/nar/gkaf370 (PMC12230705; doi:10.1093/nar/gkaf370)
Supplement: gkaf370_Supplemental_Files [file gkaf370_supplemental_files.zip › Supplementary Material 4.pdf]

Supplementary Material 4: Defining Figure 1c

The following text describes how to create the MVS state of the membrane orientation example presented in Figure 1c. The code snippets are written in Python and use the builder interface provided in the MolViewSpec library.

As with the previous example, we first need to create a MVS builder, download the structure file, apply parsing, and load the model structure. Additionally, two circles are added to the scene, which represent the membrane orientation of this membrane protein.

This example assumes the membrane boundaries are known. Mol* can predict them, and you can obtain these results using its Membrane Server CLI entry point. Start the server using:

```
Unset
node lib/commonjs/servers/membrane-orientation/server.js
```

By default, it will listen on port 1340. A simple prediction looks like this:

```
Unset
http://localhost:1340/MembraneServer/predict/3sn6/?assemblyId=1
```

Change the entry_id as needed. The server will respond with JSON, describing key values needed to draw both membrane primitives:

```python
Python
import molviewspec as mvs


server_response = {
    "planePoint1": [27.6286077232155, 10.3137003539375, 17.3841276600337],
    "planePoint2": [24.2923627786858, 13.70617189513, -17.3918785297573],
    "normalVector": [0.0950497135193607, -0.096651610860181, 0.99076940711652],
    "centroid": [25.9604852509506, 12.0099361245337, -0.00387543486177577],
    "radius": 29.8063842867283,
}
```

Additional data manipulation allows transforming this representation into values that can be consumed by the MolViewSpec builder:

```python
normal = _normalize(server_response["normalVector"])
reference = [1, 0, 0] if abs(_dot(normal, [1, 0, 0])) < 0.9 else [0, 1, 0]
major_axis = _normalize(_cross(normal, reference))
minor_axis = _normalize(_cross(normal, major_axis))

def _normalize(v):
    length = (v[0] ** 2 + v[1] ** 2 + v[2] ** 2) ** 0.5
    return [v[0] / length, v[1] / length, v[2] / length] if length != 0 else v

def _dot(a, b):
    return a[0] * b[0] + a[1] * b[1] + a[2] * b[2]

def _cross(a, b):
    return [a[1] * b[2] - a[2] * b[1], a[2] * b[0] - a[0] * b[2], a[0] * b[1] -
a[1] * b[0]]
```

With this ground work done, we can now make use of the MolViewSpec Python builder to load the 3D structure of PDB ID 3sn6, using the similar instructions as in the previous examples. Note that this is a multi-chain complex, which benefits from coloring individual chains with distinct colors. The selection of these colors can be delegated to the Mol* viewer by providing a `custom` parameter and by setting its “molstar_use_default_coloring” to true. Custom properties are supported on all MolViewSpec nodes and can be used to hold arbitrary data that is not part of the MolViewSpec schema. A collection of Mol*-specific properties allow customizing the scene though only the Mol* viewer will understand and honor these instructions:

```python
builder = mvs.create_builder()
(
    builder.download(url="https://files.wwpdb.org/download/3sn6.cif")
    .parse(format="mmcif")
    .assembly_structure(assembly_id="1")
    .component()
    .representation(type="cartoon")
    # must provide an arbitrary color to set custom properties
```

```
    .color(color="white", custom={"molstar_use_default_coloring": True})
)
```

With that, the protein will render in cartoon representation and each of its chains would be colored distinctly. To add geometric primitives for both membrane boundaries, we make use of the `.primitives()` function. This function grants access to a part of the builder that is tailored to low-level geometric shapes such as arrows, tubes, spheres etc. At the same time, it allows setting global properties that will affect all primitives grouped together by subsequent calls. Here, we adjust the `opacity` value, ensuring that the drawn membrane circles appear semi-transparent:

```python
(
    builder.primitives(tooltip="Membrane Layer", opacity=0.66)
    .ellipse(
        center=server_response["planePoint1"],
        major_axis=major_axis,
        minor_axis=minor_axis,
        radius_major=server_response["radius"],
        as_circle=True,
        tooltip="Inner Membrane",
    )
    .ellipse(
        center=server_response["planePoint2"],
        major_axis=major_axis,
        minor_axis=minor_axis,
        radius_major=server_response["radius"],
        as_circle=True,
        tooltip="Outer Membrane",
    )
)
```

Two ellipses are added to the scene, one for the inner membrane boundary, one for the outer one. The previously obtained values are provided as function arguments. The center points of these disks and their radius are directly usable from the Membrane Server response. As `major_axis` and `minor_axis`, the prepared vectors are provided. The `as_circle` flag is relevant for this use case. Alternatively, `radius_minor` can be specified to adjust the shape.

```python
builder.save_state(destination='1c.mvsj', indent=2)
```

Is the final instruction and exports this state.
