# Supplementary material for: MolViewSpec: a Mol* extension for describing and sharing molecular visualizations
Source: Nucleic Acids Res. 2025 May 6;53(W1):W408–14. doi: 10.1093/nar/gkaf370 (PMC12230705; doi:10.1093/nar/gkaf370)
Supplement: gkaf370_Supplemental_Files [file gkaf370_supplemental_files.zip › Supplementary Material 5.pdf]

Supplementary Material 5: Defining Figure 1d

The following text describes how to create the MVS state that visualizes electron density data, presented in Figure 1d. The code snippets are written in Python and use the builder interface provided in the MolViewSpec library.

As the first step, relevant 3D structure data is downloaded, parsed, and used to create a standard cartoon representation:

```python
import molviewspec as mvs

builder = mvs.create_builder()

structure =
builder.download(url="https://files.wwpdb.org/download/1tqn.cif").parse(format=
"mmcif").model_structure()
structure.component(selector="polymer").representation(type="cartoon").color(co
lor="white")
```

Next, the ligand is selected using the “ligand” selector that will match all ligands of a structure. The created component is represented in ball-and-stick style. A custom property is specified to instruct Mol* to color non-carbon atoms according to their CPK coloring. The focus command controls the viewpoint. These values can also be omitted, allowing Mol* to determine a reasonable camera position automatically:

```python
ligand = structure.component(selector="ligand")
ligand.representation(type="ball_and_stick").color(custom={"molstar_color_theme
_name": "element-symbol"})
ligand.focus(up=[0.98, -0.19, 0], direction=[-28.47, -17.66, -16.32],
radius=14, radius_extent=5)
```

The Mol* Volume Server is a convenient way to obtain electron density data efficiently. Here, we make use of a PDBe-hosted service and request density information for PDB ID 1tqn. The

provided coordinates narrow down the transferred data to a box around the ligand focused above.
MolViewSpec interacts with density data in a way that resembles steps needed for standard 3D structure data. A resource is downloaded and parsed by providing a suitable format:

```python
volume_data = builder.download(

url="https://www.ebi.ac.uk/pdbe/densities/x-ray/1tqn/box/-22.367,-33.367,-21.634/-7.106,-10.042,-0.937?detail=3"
).parse(format="bcif")
```

The obtained `volume_data` is then used to select specific channels. Dedicated representations can be added for each channel. The first block creates a visualization of the 2FO-FC electron density map, a commonly used representation in crystallography for validating model structures. An isosurface representation will show a 3D contour that represents a certain density level (configurable using the `relative_isovalue`). Visuals can be adjusted using `show_wireframe` and `show_faces`. Analogous to the representations before, the volume representation can be colored as needed. A reduced opacity ensures that other representations are visible behind the requested wireframe.

```python
volume_data.volume(channel_id="2FO-FC").representation(
    type="isosurface",
    relative_isovalue=1.5,
    show_wireframe=True,
    show_faces=False,
).color(color="blue").opacity(opacity=0.3)
```

Next, the FO-FC difference density map is retrieved. This map highlights discrepancies between observed and model-calculated densities and is useful for identifying missing atoms, alternate conformations, or errors in model building.
Two representations are created, differing only in their cutoff value and color. The first, green representation will indicate regions with a surplus in electron density where atoms might be missing in the model. The second, red representation captures missing electron density where model atoms may be incorrectly placed:

Python

```
fo_fc = volume_data.volume(channel_id="FO-FC")
fo_fc.representation(type="isosurface", relative_isovalue=3,
show_wireframe=True).color(color="green").opacity(
    opacity=0.3
)
fo_fc.representation(type="isosurface", relative_isovalue=-3,
show_wireframe=True).color(color="red").opacity(
    opacity=0.3
)
```

Finally, the generated state is saved to a file:

Python

```
builder.save_state(destination='1d.mvsj', indent=2)
```
