# Supplementary material for: MolViewSpec: a Mol* extension for describing and sharing molecular visualizations
Source: Nucleic Acids Res. 2025 May 6;53(W1):W408–14. doi: 10.1093/nar/gkaf370 (PMC12230705; doi:10.1093/nar/gkaf370)
Supplement: gkaf370_Supplemental_Files [file gkaf370_supplemental_files.zip › Supplementary Material 6.pdf]

Supplementary Material 6: Example scripts and output files

The file ‘2025-molviewspec-supplementary.zip’ contains all scripts and their expected outputs (i.e. ‘1a.mvsj’, ‘1b.mvsj’, and ‘1b.mvsx’) from Supplementary Materials 2, 3, 4, and 5. It also contains the MVS annotation file required for Figure 1b (i.e., ‘confidence-Q868N5.cif’) from the article that is referenced in Supplementary Material 3. The archive also contains snapshots of the Mol* (v4.12.1) and MolViewSpec (v1.2.1) projects as well as the accompanying Google Colab notebook at the time of writing.
