# Supplementary material for: MolViewSpec: a Mol* extension for describing and sharing molecular visualizations
Source: Nucleic Acids Res. 2025 May 6;53(W1):W408–14. doi: 10.1093/nar/gkaf370 (PMC12230705; doi:10.1093/nar/gkaf370)
Supplement: gkaf370_Supplemental_Files [file gkaf370_supplemental_files.zip › 2025-molviewspec-supplementary.zip!/2025-molviewspec-supplementary/supplementary/molstar-v4.12.1.zip!/molstar-master/docs/docs/extensions/mvs/integration-examples.html]

# Integration of Mol\* with MolViewSpec Extension

This page demonstrates several methods to integrate Mol\* Viewer in a web page and use MolViewSpec functionality.
See the source HTML to see the actual code.

## Method 1: Get MVS view from a server and pass to the viewer

The recommended method is to serve the MVS view files by your server (either as static files or generated by the
server on-demand) and call the `loadMvsFromUrl` method to retrieve and load them.
This example uses a MVS view file from the address specified in the `sourceUrl` variable.
If the MVS view file contains relative references, they will be resolved as relative to `sourceUrl`.

A variation of this method uses `molstar.PluginExtensions.mvs.loadMVS` instead of
`loadMvsFromUrl` and allows replacing the MVS view after it has been loaded.

View 1
View 2

## Method 2: Construct MVS view on frontend and pass to the viewer

Another option is to utilize the MVS builder provided by the extension to build the view on frontend and then
pass it to the viewer. This example builds the view in plain JavaScript, directly in a <script> tag in
HTML. However, for a better developer experience consider writing the code in TypeScript.
If the built MVS view contains relative references, they will be resolved as relative to the URL of this HTML
page.

Again, there is variation with using `molstar.PluginExtensions.mvs.loadMVS` instead of
`loadMvsData`.
