# Supplementary material for: MolViewSpec: a Mol* extension for describing and sharing molecular visualizations
Source: Nucleic Acids Res. 2025 May 6;53(W1):W408–14. doi: 10.1093/nar/gkaf370 (PMC12230705; doi:10.1093/nar/gkaf370)
Supplement: gkaf370_Supplemental_Files [file gkaf370_supplemental_files.zip › 2025-molviewspec-supplementary.zip!/2025-molviewspec-supplementary/supplementary/molstar-v4.12.1.zip!/molstar-master/src/examples/lighting/index.html]

Mol\* Lighting Demo
